# Ultra‐Conformable Ionic Skin with Multi‐Modal Sensing, Broad‐Spectrum Antimicrobial and Regenerative Capabilities for Smart and Expedited Wound Care

**DOI:** 10.1002/advs.202004627

**Published:** 2021-02-15

**Authors:** Xiao Lin, Yuxuan Mao, Peng Li, Yanjie Bai, Tao Chen, Kang Wu, Dandan Chen, Huilin Yang, Lei Yang

**Affiliations:** ^1^ Orthopedic Institute and Department of Orthopedics The First Affiliated Hospital, Soochow University Suzhou Jiangsu 215006 P. R. China; ^2^ School of Public Health Soochow University Suzhou 215123 P. R. China; ^3^ Jiangsu Provincial Key Laboratory of Advanced Robotics, School of Mechanical and Electric Engineering Soochow University Suzhou 215123 P. R. China; ^4^ National Institute for Food and Drug Control Beijing 102629 P. R. China; ^5^ Center for Health Science and Engineering (CHSE), School of Materials Science and Engineering Hebei University of Technology Tianjin 300130 P. R. China; ^6^ Tianjin Key Laboratory of Spine and Spinal Cord Tianjin Medical University General Hospital Tianjin 300130 P. R. China

**Keywords:** antimicrobial, gel‐point adhesive hydrogel (GPAH), ionic skin, smart wound care, tactile sensing

## Abstract

While rapid wound healing is essential yet challenging, there is also an unmet need for functional restoration of sensation. Inspired by natural skin, an ultra‐conformable, adhesive multi‐functional ionic skin (MiS) with multi‐modal sensing capability is devised for smart and expedited wound care. The base of MiS is a unique skin‐like, conductive and self‐adaptive adhesive polyacrylamide/starch double‐network hydrogel (PSH) and self‐powered, flexible, triboelectric sensor(s) is integrated on top of PSH for multi‐tactile sensing. MiS could enhance wound contraction, collagen deposition, angiogenesis, and epidermis formation in a full‐thickness skin defect wound model *in vivo*, while significantly inhibiting the biofilm formation of a wide range of microorganisms. MiS also exhibits multi‐modal sensing capability for smart and instant therapeutics and diagnostics, including skin displacement or joint motion, temperature, pressure and tissue exudate changes of wound bed, and locally releasing drugs in a pH‐responsive manner. More importantly, MiS could restore the skin‐mimicking tactile sensing function of both touch location and intensity, and thus could be used as a human‐machine interface for accurate external robotic control. MiS demonstrates a new comprehensive paradigm of combining wound diagnosis and healing, broad‐spectrum anti‐microbial capability and restoration of multi‐tactile sensing for the reparation of severe wound.

## Graphical abstract

An ultra‐conformable, adhesive multi‐functional ionic skin (MiS) with multi‐modal sensing capability is devised for smart and expedited wound care. MiS is composed of a polyacrylamide/starch double‐network hydrogel and a self‐powered, flexible, triboelectric sensor. MiS demonstrates a comprehensive paradigm of combining wound diagnosis and healing, broad‐spectrum anti‐microbial capability and restoration of multi‐tactile sensing for advanced wound management.

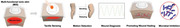

## 1 Introduction

Severe wounds caused by trauma or diseases include the damage of skin and various organs or tissues and loss of their biological functions, influencing 2 to 4.5 million people and costing $25 billion per year for treatment in the US only.^[^ ^1^ ^]^ To date, repair of tissue injury as well as restoration of key functions remains a great challenge, and wound infection is still a formidable problem. Skin senses multi‐tactile stimuli with high sensitivity, protects the body from damage and microbial invasion, and regulates the homeostasis of body temperature, electrolytes, and nutritional components in a stimuli‐responsive manner.^[^ ^2^, ^3^, ^4^ ^]^ Severe skin damage or chronic wound leads to the loss of protective and regulatory functions, disturbs the repair of damaged tissue underneath, and often causes infection or other secondary tissue damages at time periods lasting for up to months or even years.^[^ ^5^, ^6^ ^]^ In addition, severe skin damage causes temporary (from days to months) or permanent sensory losses that impair tactile, thermal and biomechanical sensations during or after wound healing process. Therefore, a comprehensive solution to wound healing, including at least promoted tissue healing, prevention of infection, instant monitoring of wound conditions, spontaneous on‐demand medication and restoration of multi‐tactile sensation, is still lacking. Current wound care materials and dressings focus solely on managing the wound healing process and can neither monitor wound condition nor restore sensation function.^[^ ^7^, ^8^, ^9^, ^10^ ^]^ Emerging flexible integrated electronics with tactile sensibility enable possible solutions to sensory disorders, potentially allowing patients to monitor wound condition and regain tactile sensing capability.^[^ ^11^, ^12^, ^13^ ^]^ Nevertheless, current integrated electronics have rarely been used in wound management due to lack of biocompatibility to the wounds and poor wound healing capability. Recently, hydrogel‐based ionic skins^[^ ^14^, ^15^, ^16^, ^17^ ^]^ showed great wound healing potential as well as unique electrical and/or environment responsive properties for achieving the requirements of smart wound care,^[^ ^7^, ^18^, ^19^ ^]^ opening a new route to address the challenges in the cure for severe skin damage. Here, we developed an ultra‐conformable, hydrogel‐based ionic skin that highly mimics the properties, biological functions, and multi‐modal sensation of natural skin for smart and expedited wound care. The ionic skin exhibits broad‐spectrum antimicrobial, pro‐regenerative, and multi‐modal sensing capabilities for smart and expedited cure of full‐thickness skin wound.

**Figure 1:**
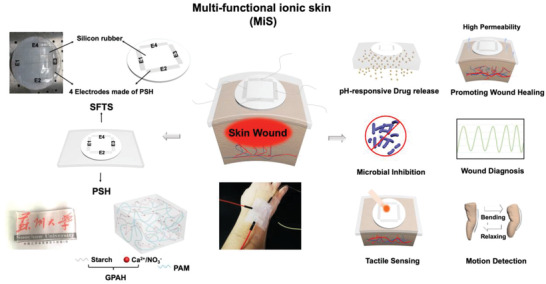
Schematic illustration of the structure of the ultra‐conformable and adhesive multi‐functional ionic skin (MiS), and its application for smart and expedited wound care through promoting wound healing, preventing infection, diagnosing wound conditions (temperature, pressure, exudation), and sensing complex tactile signals. GPAH: gel‐point adhesive hydrogel; PAM: polyacrylamide; SFTS: self‐powered, flexible, triboelectric sensor.

Inspired by the natural skin, we devised an ultra‐conformable, adhesive multi‐functional ionic skin (MiS), which could realize multi‐tactile sensing and smart wound healing management, simultaneously (**Figure** **1**). A starch‐based, gel‐point adhesive hydrogel (GPAH) was developed as a functional component and incorporated with base polymers to constitute the functional components of MiS, enabling a number of key functions for smart wound care. Specifically, in order to optimize the interface between MiS and the wound bed, a double‐network hydrogel as the base of MiS was developed through modifying polyacrylamide (PAM) with GPAH (hereafter referred to as PSH). PSH exhibits the abilities to maintain a moist wound environment, absorb excess tissue exudate, allow air to permeate and exchange at the wound surface, and be easily deployed to or removed from the wound bed before and after treatment thanks to a self‐adaptive adhesiveness, all of which are essential for expedited wound healing.^[^ ^20^, ^21^ ^]^ Besides, PSH possesses skin‐matched mechanical and electrical properties, thermal and biomechanical sensations, as well as efficient broad‐spectrum antimicrobial activity, while maintaining a high degree of biocompatibility. Inspired by our previous study,^[^ ^22^ ^]^ a self‐powered, flexible, triboelectric sensor (SFTS) that consists of PSH flexible electrodes and a Ecoflex/polyethylene substrate was integrated on the top of PSH for multi‐tactile sensing. The PSH base and SFTS of MiS are wire‐connected with signal acquisition devices for detection of various signals in the present work, but MiS certainly can be further upgraded via wireless technologies.^[^ ^23^, ^24^ ^]^

MiS shows great potential for smart wound care by enabling pro‐regenerative potential for full‐thickness skin defect wounds, wound diagnosis via monitoring of joint or skin motion and displacement, temperature, tissue exudation and pressure at the wound site, and smart therapeutic capability through pH‐responsive drug release, as well as detection of both location and intensity of a touch that can be further used to control external robotic arm. MiS overcomes several limitations associated with the application of traditional wound dressing materials by realizing an ionic skin for smart wound care, which provides a robust tissue anchor, assures high conformability to native skin and high biocompatibility, possesses efficient antimicrobial properties, promotes wound regeneration, and has wound‐monitoring ability.^[^ ^3^, ^25^, ^26^ ^]^

## 2 Results and Discussion

### 2.1 Physical and Chemical Properties of the PSH for Smart Wound Care

The PSH in MiS, which is responsible for wound healing and wound site sensing, is a hybrid network of chemically cross‐linked PAM and ionically cross‐linked GPAH,^[^ ^27^ ^]^ resulting in both self‐adaptive adhesiveness onto various surfaces and strong cohesion within the network. PAM is known for high swelling capability and relatively balanced strength and flexibility, whereas GPAH developed in our previous studies has unique viscoelastic properties, high stretchability, tissue or skin adhesiveness, and moisture retention ability.^[^ ^27^ ^]^ GPAH provides abundant hydroxyl groups and numerous hydrogen bond donors and acceptors to form a hydrophilic network, which could dramatically modify the physical and chemical properties of PAM. Fourier transform infrared (FTIR) analysis of PSH chemical structure (Figure S1, Supporting Information) indicates no chemical reaction between PAM and GPAH, and that the two components interacted physically. Although the addition of GPAH inevitably decreased the cross‐linking density of PAM (see the analysis in the Supporting Information), PAM and GPAH formed a uniform, interpenetrating network, and the resultant PSH was homogeneous, transparent and had no visible sign of phase separation (photographs in Figure 1 and Figure S2: Supporting Information).

**Figure 2:**
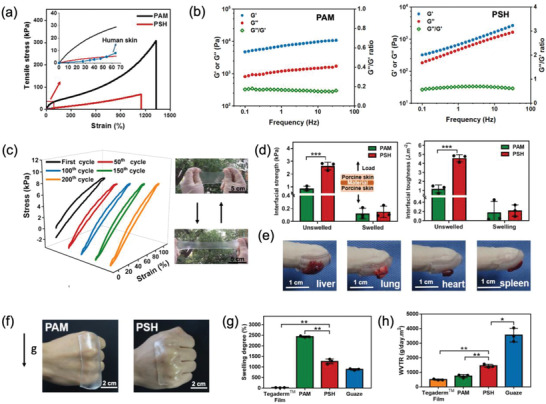
Physical and chemical properties of PSH in MiS. a) Stress–strain curves of PSH, PAM, in comparison to human skin.^[^ ^29^ ^]^ b) Dependence of *G*′, *G*′′, and the *G*′′/*G*′ ratio (loss factor) of hydrogels on the frequency of oscillation. c) Photos and stress–strain curves of the PSH at different stretching cycles up to 200 cycles. Tests were performed at a maximum strain of 100% under the displacement rate of 100 mm min^−1^. d) Material/porcine skin interfacial strength and toughness before and after swelling, indicating a self‐adaptive adhesiveness of PSH. Data = mean±standard deviation (*n* = 3). ***p < 0.001 by two‐tailed unpaired Student's *t*‐test. e) PSH firmly adhered to various wet tissues. f) PAM and PSH film adhering on a human palm joint. Compared with PAM, PSH could form a highly conformable and tight contact with irregularly contoured skin. g) Swelling degree of various materials. Data = mean±standard deviation (*n* = 3). **p < 0.01 compared with the PSH group by two‐tailed unpaired Student's *t*‐test. h) Water‐vapor transmission rate (WVTR) of various materials. Data = mean±standard deviation (*n* = 3). *p < 0.05, **p < 0.01 compared with the PSH group by two‐tailed unpaired Student's *t*‐test. Thickness of Tegaderm film = 0.1 mm.

The PSH owns a number of mechanical, physiochemical, and electrical properties that either match those of native skin or possess diagnostic and therapeutic promise for smart wound care. The PSH is robust but highly flexible and adhesive, able to form a tightly conformal anchor to curvilinear and dynamic wound surfaces. The addition of GPAH to PAM endowed the resultant PSH with a tensile behavior close to that of native human skin (**Figure** **2a**),^[^ ^28^, ^29^ ^]^ especially when the applied strain was less than 50%, which is a strain range involved in most human motions, such as bending of the knees, finger joints, and wrists.^[^ ^30^, ^31^ ^]^ Additionally, PSH as a hybrid cross‐linked network showed much higher loss factor (tan *δ* ≈0.7) relative to that of the covalently cross‐linked PAM (tan *δ* ≈0.2) (Figure 2b) which is due to the gel‐point viscoelasticity of GAPH (i.e., tan *δ* ≈1).^[^ ^27^ ^]^ Interestingly, PSH has a storage modulus comparable to that of native skin (Figure S3, Supporting Information) but exhibited a higher loss factor (tan *δ* ≈0.2 for native skin).^[^ ^32^ ^]^ The resultant high‐loss factor viscoelastic behavior of PSH indicates an improved adaption to skin deformation when it adhered to the wound site.^[^ ^17^ ^]^ Importantly, the stress‐strain correlation of PSH remained nearly unchanged after hundreds of loading/unloading cycles (Figure 2c), suggesting its reliability when applied to places like joints that undergo frequent motion. Overall, PSH displayed a good combination of mechanical robustness, flexibility, and viscoelastic characteristics enabling adaption and toleration to skin or joint deformation.

We then tested hydrogel adhesiveness to porcine skin (which is highly similar to human skin), using pull‐off adhesion tests. As shown in Figure 2d the interfacial strength and toughness of the PSH (2.63 ± 0.30 kPa and 4.59 ± 0.39 J m^−2^) were significantly higher than those of PAM (0.88 ± 0.17 kPa and 1.18 ± 0.44 J m^−2^), enabling PSH to easily adhere and anchor onto skin and wet tissues (Figure 2e,f). In fact, the tissue adhesiveness of MiS is at the same level with recently developed silk fibroin adhesives,^[^ ^33^ ^]^ adhesive skin patch,^[^ ^34^ ^]^ and adhesive hydrogel wound dressings.^[^ ^3^, ^35^, ^36^, ^37^ ^]^ The greatly improved adhesiveness of PSH is attributed to the large amount of hydrogen bonds formed between tissue amino groups and hydroxyl groups from GPAH,^[^ ^38^, ^39^ ^]^ and to the high‐loss factor viscoelasticity that allows an increased contact area with microrough skin surfaces^[^ ^40^ ^]^ and increased energy dissipation due to plastic re‐arrangement of starch network during detaching.^[^ ^41^ ^]^ The combination of good tissue adhesiveness and mechanical flexibility allows PSH to conformably and tightly contact irregularly contoured human skin (Figure 2f), which would be important for reliable wound healing and diagnosis.

More importantly, the adhesiveness of PSH is self‐adaptive to the wetness of wound bed and varies depending on adsorption of wound exudate (Figure 2d). Since wound care materials need to be removed and replaced periodically, secondary injury always occurs during the removal process due to tissue‐dressing adhesion. For example, gauzes usually adhere to the wound bed because of the infiltration of wound exudate into the gauzes, resulting in secondary injury by damaging newly formed tissue during dressing replacements.^[^ ^42^, ^43^ ^]^ When the PSH was fully swollen in deionized water, the interfacial strength and toughness markedly decreased to 0.15 ± 0.09 kPa and 0.22 ± 0.12 J m^−2^ (i.e., 1/18 and 1/21 of the values before swelling, respectively), respectively. This wetness‐mediated adhesiveness of PSH allows convenient replacement of MiS with reduced pain and avoiding secondary damage after absorbing exudates from the wound. In addition, the adhesiveness of MiS increased slightly (interfacial strength and toughness increased to 3.03 ± 0.91 kPa and 4.94 ± 1.98 J m^−2^, respectively) when absorbing 50 wt% of saline. This property indicates that in clinical scenario MiS would not decrease its adhesive performance when absorbs normal amount of sweat. In case of prolonged perspiration, significant decrease in adhesiveness would be less likely to happen due to the balanced absorption and transmission of sweat (insensible perspiration rate is less than 1300 g m^−2^ d^−1^ at rest,^[^ ^44^ ^]^ which is lower than the water‐vapor transmission rate of MiS described later). However, exposure to large amount of fluids from the ambient environment (like immersed in water) should be avoided since it may substantially decrease the adhesiveness.

The swelling degree of the PSH was 1260 ± 115% (Figure 2g), indicating that MiS has an exudate‐absorbing capability similar to that of the widely used medical gauze (swelling degree was 884 ± 38%). Besides, the PSH has a proper water vapor permeability (Figure 2h and Table S1: Supporting Information), which is a key factor to ensure a suitable moisture content at the wound bed for wound healing.^[^ ^20^ ^]^ The water‐vapor transmission rate (WVTR) of PSH was 1442 ± 111 g m^−2^ day, about 41% of that of medical gauze (3548 ± 472 g m^−2^ day). Compared to PAM (740 ± 110 g m^−2^ day) and several commercially available wound care products such as Honey Pads (700 g m^−2^ day), Scar Away (255 g m^−2^ day), and Tegaderm film dressing (495 ± 41 g m^−2^ day), PSH had much higher water vapor permeability that is favorable for granulated tissue growth^[^ ^20^ ^]^ and for creating an oxygen‐enriched environment within the wound bed.

The PSH is also an electrically conductive, self‐healing hydrogel with electrical conductivity of 0.475 mS cm^−1^, which is similar to that of native skin (between 2.6 and 10^−4^ mS cm^−1^ depending on different skin components^[^ ^45^ ^]^). Damaged PSH could completely recover its electrical conductivity to the level of intact one when the damaged edges were simply re‐contacted (Figure S4, Supporting Information), which should be due to perfect physical contact between edges and the rapid self‐healing capability of GPAH network within PSH.^[^ ^46^ ^]^ Although the normal insensible perspiration will not cause the variation in water content of the PSH due to a high WVTR of PSH compared to insensible perspiration rate, the perspiration could lead to NaCl retention in the PSH. Interestingly, a simulation test showed that the conductivity of the PSH had a minimal change when adding 0.9 wt% NaCl in the matrix (0.495 mS cm^−1^), indicating that the perspiration‐caused NaCl retention in PSH would not change the electrical property of the MiS. Conductively robust, stable and self‐healing PSH allows further design of MiS that can convert physiological signals at the wound site into reliable electrical signals for multi‐modal sensing of wound conditions.

### 2.2 *In Vitro* Biocompatibility and Antimicrobial Capabilities of MiS

**Figure 3:**
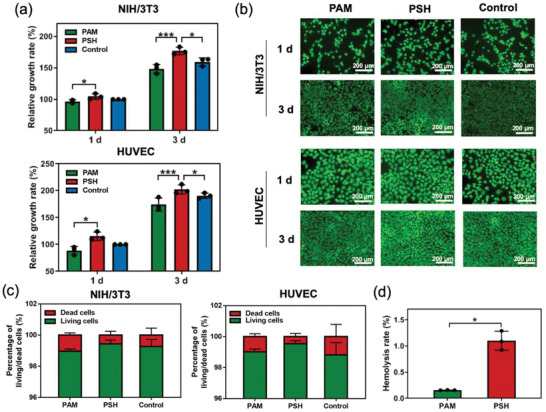
*In vitro* cell and blood compatibility of PSH in MiS. a) Relative growth rates of NIH/3T3 fibroblasts and HUVEC when cultured with the hydrogel extracts for 1 and 3 days, versus a control group at day 1 (set as 100%). Data = mean±standard deviation (*n* = 3). *p < 0.05, ***p < 0.001 compared with the PSH group by two‐tailed unpaired Student's *t*‐test. b) Fluorescence micrographs of Live/Dead stained NIH/3T3 fibroblasts and HUVEC after being cultured with the hydrogel extracts for 1 and 3 days. c) Percentage of living/dead NIH/3T3 fibroblasts or HUVEC when cultured with the hydrogel extracts for 1 day. Data = mean±standard deviation (*n* = 3). d) Hemolysis rate of hydrogel extracts. Data = mean±standard deviation (*n* = 3). *p < 0.05 by two‐tailed unpaired Student's *t*‐test.

MiS demonstrated high cell and blood compatibility and antimicrobial properties for wound healing applications. The extract of PSH (the tissue‐contacting part of MiS) supported the proliferation of NIH/3T3 fibroblasts and human umbilical vein endothelial cells (HUVEC) after 1 and 3 days of culture as compared with the use of culture medium alone (control group) (**Figure** **3** **a**). In contrast, proliferation of both cell types grown in PAM extract was suppressed relative to that in the control group. The high cyto‐compatibility of PSH was further confirmed by Live/Dead staining of cells (Figure 3b,c), revealing higher density of viable cells in the PSH group than in the control group along with normal cell morphology. In addition, the hemolysis rate of PSH was <1.5% (Figure 3d), and this suggests no hemolysis risk and a high degree of blood compatibility according to the ASTM standard F‐756‐08. Other biocompatibility tests *in vivo* such as skin irritation test will be evaluated in the future study.

**Figure 4:**
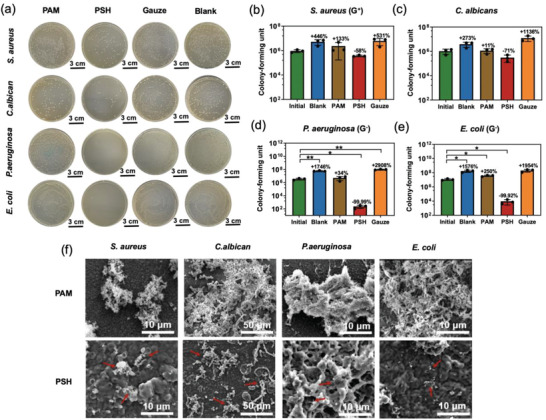
*In vitro* broad‐spectrum antimicrobial capabilities of PSH in MiS. a) Representative images of viable colonies of *S. aureus*, *C. albicans*, *P. aeruginosa*, and *E. coli* grown on agar plates after contacting with different materials. b–e) Quantification of viable *S. aureus*, *C. albicans*, *P. aeruginosa*, *E. coli* and the corresponding antibacterial ratios after contacting with different materials. Data = mean±standard deviation (*n* = 3). *p < 0.05, **p < 0.01 compared with the initial group by two‐tailed unpaired Student's *t*‐test. f) SEM images of *S. aureus*, *C. albicans*, *P. aeruginosa*, and *E. coli* biofilms after cultured on PAM and PSH for 4 h. Red arrows indicate sparse microbes on PSH.

A challenging problem of hydrogel‐based wound care materials is that they easily adsorb microorganisms, which further form biofilms that are difficult to eradicate,^[^ ^47^ ^]^ causing severe wound infections that impede the healing process and even life‐threatening complications. We evaluated the broad antimicrobial properties of PSH against Gram‐negative (*E. coli*) and Gram‐positive (*S. aureus*) bacteria as well as biofilm‐forming bacteria (*S. aureus* and *P. aeruginosa*) and fungus (*C. albicans*) (**Figure** **4**), all of which are involved in the common infections of both acute and chronic wounds.^[^ ^48^, ^49^, ^50^, ^51^, ^52^, ^53^, ^54^, ^55^, ^56^ ^]^ The PSH in MiS, which directly contacts with wound bed, showed much stronger inhibitory effects on all tested microbes than control samples including medical gauze and PAM (Figure 4a). PSH killed 99.9% of *E. coli* and *P. aeruginosa*, 58% of *S. aureus*, and 71% of *C. albicans* (Figure 4b–e) in the microbial contact tests. SEM observation (Figure 4f) showed adherence of significantly fewer and severely deformed microbes to PSH as compared with PAM. Also, biofilms of all four types of microbes were formed on PAM, but there was no biofilm formation on PSH (Figure 4f). These results indicate a strong and possibly broad‐spectrum inhibitory activity of PSH against the microbes. This inhibitory activity was possibly attributed to the nitrate ions in PSH since nitrate could be converted to nitrite through bacterial reduction, which could further be reduced to NO (known for its potent antibacterial effects) under the abundant supply of protons by PSH,^[^ ^57^, ^58^ ^]^ as well as to the softness of PSH, known to inhibit adherence of microbes.^[^ ^59^, ^60^, ^61^, ^62^ ^]^ The proposed mechanisms are based on the fact that PSH is softer than PAM and contains nitrate ions. MiS therefore has a high likelihood to avoid side effects associated with inappropriate use of antibiotics^[^ ^63^, ^64^ ^]^ and the potential toxic effects of antimicrobial peptides or nanoparticles.^[^ ^65^, ^66^, ^67^ ^]^ The PSH in MiS simultaneously demonstrated good cell and blood compatibility, as well as efficient broad‐spectrum antimicrobial effects, thereby providing a solid basis for using MiS in wound healing management applications.

### 2.3 Expedited Healing of Full‐Thickness Skin Defect Wound with the MiS

**Figure 5:**
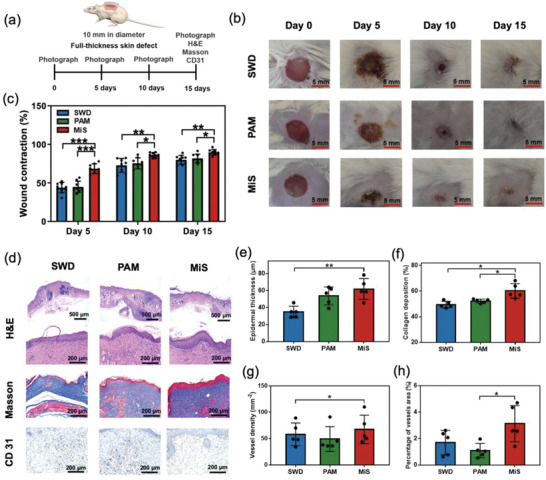
Healing of murine full‐thickness skin defect wound with the MiS. a) Schematic illustration of the experimental design. b) Representative images of the skin wounds after treated with commercial sterile wound dressing (SWD), PAM, and MiS for 5, 10, and 15 days. c) Wound contraction ratios of different treatment groups. Data = mean±standard deviation (*n* = 8). *p < 0.05, **p < 0.01, ***p < 0.001 compared with the MiS group by two‐tailed unpaired Student's *t*‐test. d) Representative images of H&E, Masson, and CD31 immunohistochemical stainings of wound tissues treated with SWD, PAM and MiS at 15 days post‐surgery. e) Epidermal thickness, f) collagen deposition, g) vessel density, and h) percentage of vessel area at the wound site after different treatments for 15 days. Data = mean±standard deviation (*n* = 5). *p < 0.05, **p < 0.01 compared with the MiS group by two‐tailed unpaired Student's *t*‐test.

A murine full‐thickness skin defect wound model was created to evaluate the effect of the MiS on wound healing and its progress at different time points (**Figure** **5** **a**). Representative photographs of the healing process of skin defects in the groups of MiS, PAM, and clinically used sterile wound dressing are shown in Figure 5b. Statistical measurement results based on wound contraction progress (Figure 5c) suggest that the wound healing rate of the MiS group was significantly higher than that of the PAM and sterile wound dressing groups at all three observation time points, especially at the early stage (<10 days). The healing rate of the MiS treated wound (≈69% at day 5, ≈86% at day 10, and 90% at day 15) is comparable or even higher than other reported wound healing materials, such as chitosan/silk fibroin cryogel (≈60% at day 7 and ≈90% at day 15 for *φ*8 mm full‐thickness skin wounds in mice),^[^ ^68^ ^]^ chitosan‐based hydrogel (≈65% at day 5 and ≈86% at day 10 for *φ*7 mm full‐thickness skin wounds in mice),^[^ ^37^ ^]^ or a polypeptide‐based hybrid nanosystems (≈67% at day 7 and ≈92% at day 10 for *φ*10 mm full‐thickness skin wounds in mice).^[^ ^69^ ^]^ H&E staining for histologic analysis of tissues retrieved at 15 days post‐surgery revealed that the MiS‐treated group had denser tissue structures relative to those from the PAM and sterile wound dressing groups (Figure 5d). The epidermal thickness (Figure 5d,e) in the MiS group was significantly higher than that of the other two groups. During maturation stage of wound repair, wound contraction occurs when fibroblasts cross‐linked with collagen.^[^ ^70^ ^]^ The Masson trichrome staining assays on the deposition of nascent collagen in the regenerated skin tissue (Figure 5d,f), which plays an important role in the physiological process of scar formation and wound contraction, also revealed that there was significantly higher collagen deposition in the MiS group (60.2 ± 5.7%) relative to that in the PAM and sterile wound dressing groups (52.2 ± 1.5% and 49.5 ± 2.5%, respectively). Additionally, Masson trichrome staining of blood vessels, which are crucial for transport of growth factors, oxygen, and nutrients to the wound bed, indicated increased neovascularization during wound healing in the MiS group relative to that in the other two groups. Further quantification of blood vessel formation by immunohistochemical staining for CD31 (Figure 5d–h) confirmed significantly higher density of newly formed blood vessels with larger mean diameter in the MiS group relative to the PAM and control groups. The results of faster wound contraction and greater collagen deposition, epidermis formation, and neovascularization in the wounds suggest the significant pro‐regenerative potential of MiS for expedited wound healing. These effects were likely attributed to: 1) the antimicrobial capacity of PSH to prevent microbe‐dependent disruption of the healing process in the early stages; 2) a moist healing environment for the wound; and 3) a favorable ionic micro‐environment at the wound bed through gradual release of Ca^2+^ ions from the PSH, which benefited wound healing as a critical regulator of epidermal homeostasis involving several signaling cascades critical to wound healing^[^ ^71^, ^72^, ^73^ ^]^ and accelerating skin wound closure.^[^ ^72^ ^]^ The healing response of fixed‐skinned mammals such as pig would be studied in the future to better evaluate the effect of MiS on the re‐epithelialization and granulation mediated healing process. Furthermore, considering its strong antibacterial capability, the potential of MiS in treatment of infected wound will also be investigated by infected animal model.

### 2.4 Multi‐Stimuli Responsiveness of MiS for Smart Wound Care

**Figure 6:**
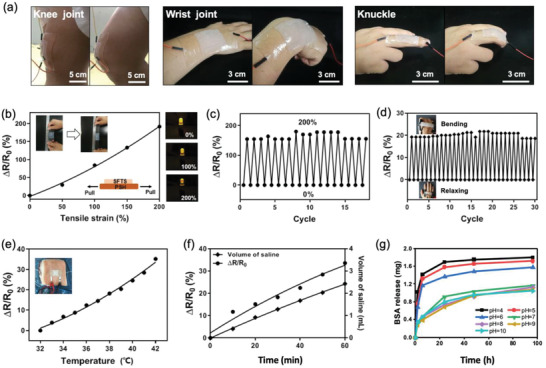
Multi‐stimuli responsiveness of the MiS for smart wound care. a) Photographs of MiS on three different joints, demonstrating high conformability. b) Variation in △*R*/*R*_0_ values in accordance to tensile strains. The variation was also demonstrated by the brightness of a LED that connected in series with MiS. Insets are photographs of a MiS before and after stretching. c) Changes of △*R*/*R*_0_ under cyclic tensile strains. d) Performance of the MiS adhered on the metacarpophalangeal joint as a strain sensor for instantly detecting metacarpophalangeal joint movements. Insets show the MiS on the hand at relaxed and bending state. e) Variation in △*R*/*R*_0_ responding to the temperature change in an *ex vivo* wound model. f) Variation in △*R*/*R*_0_ and saline volume absorbed into a MiS sample measured by an *ex vivo* wound model for 60 min at 35 ℃. The volume of PSH base of MiS is 1 cm^3^ and the contact area is 5 cm^2^. g) *In vitro* release of BSA from PSH when immersed in PBS at pH values of 4, 5, 6, 7, 8, 9, and 10.

MiS bears the potential for smart wound care, which includes diagnosis of wound conditions (such as severe inflammation, infection, or wound deterioration) and instant therapeutic treatment (by controlled drug release for example). **Figure** **6** **a** demonstrates that MiS could adhere on the skin at various moving joints with high conformability, which is one of the prerequisites for a reliable diagnosis of wound conditions. We tested the multi‐modal sensation of the MiS responding to various stimuli that could affect wound healing or reflect altered wound conditions. The electrical signal (relative change in resistance, △*R*/*R*_0_) generated from MiS deformation showed nearly linear response to tensile loading (or strain) on the PSH base (Figure 6b), which was also demonstrated by the brightness of a LED that connected in series with PSH base in MiS. This linearity indicates that MiS would principally monitor the pressure change under the MiS‐covered wound bed, as the wound site pressure influences the wound healing progress.^[^ ^74^, ^75^ ^]^ In addition, PSH has an elevated gauge factor at increased strains, showing the highest value at 400% strain (Figure S5, Supporting Information). Simulated stretching cyclic test were then carried out to evaluate the accuracy and reliability of MiS by measuring the pressure (strain) change (Figure 6c). The resistance of MiS revealed instant responses to the simulated cycles with high repeatability, and the correlation between strain and resistance change (△*R*/*R*_0_) remained identical across the cycles, indicating a consistent and reliable response to the pressure (strain) change.

The MiS was also attached to the back of a hand to monitor metacarpophalangeal joint movements (Figure 6d). The motion of metacarpophalangeal joints when fist clenched and opened was immediately reflected by a change in the measured resistance of MiS with high repeatability. The strain sensing capability of the MiS was tested on a rat joint with and without wound (Figure S6, Supporting Information). The PSH layer of MiS placed on the wound could detect the bending of the joint and output a signal that is consistent with what tested on the knee joint without wound. This monitoring ability enables MiS to track the motion recovery of an injured joint^[^ ^75^ ^]^ and to alert irregular skin or joint displacement under the MiS as an alarm of wound deterioration or healing failure. For sustaining long‐term retention on skin near the frequently moved joint or in a heavy perspiration condition, the interfacial toughness could be improved by referring to recently developed strategies.^[^ ^76^ ^]^ However, materials with ultra‐high wet adhesiveness may be difficult to remove from the wound, causing great pain to patient or even damage to the newly formed tissue. Material with zonally varied adhesiveness (i.e., low adhesiveness to the wound bed and high adhesiveness to the intact skin) is a potential solution to maintain stable adhesion for desired time while reduce risk of secondary damage during dressing replacement.

Elevated temperature at wound sites is a well‐established marker of infection and can be employed as an early indicator of chronic wounds.^[^ ^77^ ^]^ Test in an *ex vivo* wound model demonstrated that the resistance of MiS was responsive to temperature with a relatively high sensitivity and responsiveness,^[^ ^78^, ^79^ ^]^ with the change in resistance strongly correlated to the change in temperature (Figure 6e and Figure S7: Supporting Information). This indicated that the MiS was capable of timely monitoring the wound temperature to allow identification of emerging inflammation or infection. Moreover, the resistance of the MiS showed instant change and monotonic response when absorbing different amounts of saline (0.9% NaCl water solution) from the *ex vivo* wound model simulating the progress of wound exudation (Figure 6f and Figure S8: Supporting Information). Since NaCl addition cause little variation in conductivity of PSH, the response to saline should be ascribed to change in water content in PSH. This responsiveness of MiS renders the instant monitoring of exudate secretion from the wound bed possible. It is worth mentioning that the △*R*/*R*_0_ value changes linearly with time at relatively low temperature (35 ℃), while changes in a power law behavior at high temperature (42 ℃). Such differentiated signal characteristics could be potentially used to solve the problem of coupling and cross‐talk of various signals from different wound conditions.

Besides responsive capabilities for diagnosis, MiS may also provide a therapeutic option to wound treatment through the ability of controlled release of drugs. The PSH in MiS could release BSA, a common model drug, in a pH‐mediated manner (in a pH range of 4 to 10), and the release rate increased at low pH values, between 4 and 6 (Figure 6g). The pH‐mediated release could be attributed to two aspects. First, the swelling ratio of the PAM‐based hydrogel network decreases at lower pH levels^[^ ^80^ ^]^ as shown in Figure S9 (Supporting Information), which leads to a higher drug concentration in PSH, enhancing its release to surrounding aqueous solution due to higher concentration gradient. Second, degradation rate of starch could increase in low‐pH environment due to the acid‐catalyzed hydrolysis,^[^ ^81^, ^82^ ^]^ accelerating drug release from hydrogel. Since pH values at the wound bed with different levels of inflammation are in the acidic range,^[^ ^83^, ^84^ ^]^ anti‐inflammatory drugs could be loaded in MiS and released to the wound bed as the inflammation develops. In addition, the pH‐responsive drug release capacity of MiS could facilitate healing or regeneration of tissue.^[^ ^37^, ^85^ ^]^

The instant multi‐modal sensation of signals near the wound bed (including pressure, temperature, exudate status, joint or skin motion and displacement) by MiS could be used as direct indicators of wound status, or collectively analyzed to alarm the progression of events like inflammation or infection. The potential problem of signal coupling and cross‐talk is a key to future translational application and the multi‐modal signal detection is under investigation by analyzing subtle signal characteristics and by a modularity design of the device, which is divided into several functional subareas and each responds to a specific stimulus. MiS also exhibited antimicrobial and controlled drug release capabilities to intervene the inflammation or infection and pro‐regenerative potential to accelerate wound healing. Given these results, we presume that, in the near future, the processes of detection, analysis, and intervention or treatment by MiS would be integrated, programmed, and automated to fulfill the high level of smart wound care with improved accuracy and efficacy.

### 2.5 Multi‐Tactile Sensing Capability of MiS and its Application in External Robotic Control

**Figure 7:**
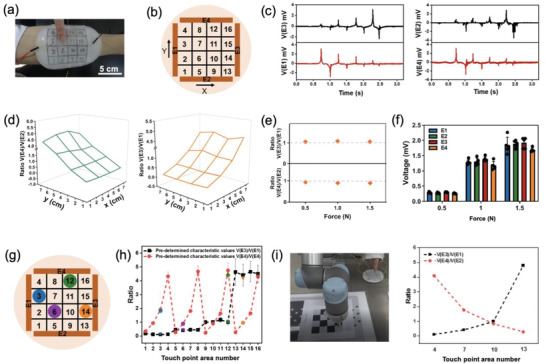
Multi‐tactile sensing capability of MiS and its application in accurate external robotic control. a) Photograph of a MiS on human skin for sensing of complex tactile signals. b) Schematic illustration of the configuration of the top surface of MiS. The area enclosed within the four electrodes was divided into 16 lattices for location recognition. c) Measured voltage signals from four electrodes when a finger touched the lattices No. 4, 7, 10, and 13 in sequence. d) Voltage ratios of E3/E1and E4/E2 (averaged from 15 independent tests) with 16 testing points. e) Voltage ratios of E3/E1, E4/E2 and f) voltages of E1, E2, E3 and E4 tested at lattice No. 11 under different forces. Data = mean ± standard deviation (*n* = 5). g) Schematic illustration of four points selected for reliability test. h) All pre‐determined characteristic V(E3)/V(E1) and V(E4)/V(E2) values (means and standard deviations of 15 independent tests) of all 16 lattices, as well as the values of lattices No. 3, 6, 12, and 14 in the reliability test. i) MiS and a robotic arm in a system for demonstrating the capability of MiS as a flexible human‐machine interface to accurately control the robot arm through tactile signals. The system included MiS, a signal‐acquisition system, a computer, a drive system, and a robotic arm. Variation in V(E3)/V(E1) and V(E4)/V(E2) values when a finger touched lattices No. 4, 7, 10, and 13 in sequence was used as input data for controlling the robotic arm.

The MiS also aims to reconstruct or replace the multi‐tactile sensing capability of the skin at the wound site, such as the detection of touch location and intensity, which has not been reported before in wound dressings. To achieve this challenging function, a strategy of integrating SFTS (**Figure** **7** **a**) with PSH was proposed. SFTS can generate voltage signals when its surface is touched, and the signals are simultaneously detected by all four strip‐like electrodes made of PSH (E1–E4 shown in Figure 7b) and voltage signals are denoted as VE1–VE4 accordingly ( Figure 7c). Theoretically, two voltage ratios from the opposite electrodes, denoted as R1 and R2 (i.e., R1 = V(E3)/V(E1) and R2 = V(E4)/V(E2)) could be used to determine the touch position on the sensor surface. While the voltage magnitude reflects the touch intensity. However, the stable signal detection of SFTS on hydrogel is a challenge. After material/device optimization, R1 and R2 of MiS revealed monotonical increase along the *X*‐ and *Y*‐axis directions, respectively, thus resulting in a unique coordinate (R1, R2) value for each touch position (Figure 7d). The tests at the same location but under different touch intensities (touch forces 0.5, 1, and 1.5 N) revealed that the voltage ratios R1 and R2 remained the same (Figure 7e), indicating that the touch intensity did not affect touch location identification. However, the magnitudes of each voltage signals changed linearly with touch intensity (Figure 7f), suggesting that MiS could differentiate the touch intensity. To verify the accuracy of the tactile sensing capability of MiS, four touch points randomly falling into 4 of 16 grids on the MiS top surface (Figure 7g), resulted in coordinate (R1, R2) values that fitted well with the pre‐determined characteristic coordinate values (Figure 7h). Meanwhile, the tactile‐sensing capability of the MiS was tested by a wound model *in vivo* (Figure S10, Supporting Information), which showed that the MiS could detect the tactile signals with a high accuracy when applied on the wound bed *in vivo*. Furthermore, the accuracy of the tactile sensing capability of MiS could be improved in the future by updating of the device design, such as adding a resin grid layer on the surface of SFTS by 3D printing.^[^ ^22^ ^]^

The multi‐tactile sensing capability of MiS unlocks new, wide opportunities to new human‐machine applications for wound care patients with severe trauma, dyskinesia, or disability. We demonstrate a preliminary robotic solution to these patients based on the MiS as a flexible human‐machine interface (Figure 7i and Movie S1: Supporting Information). The MiS could detect continuous motion and thereby track the trajectory, as continuous and trajectory touching on the top surface of MiS resulted in continuously monotonic but opposite trends in R1 and R2 values. Therefore, the instant motion and location control of a robotic arm with high accuracy was realized by touching on MiS. This demonstration suggests the potential of MiS in human‐machine applications to allow patients with dyskinesia or disability to regain movement and operating capabilities even during the stage of wound care. Meanwhile, the reusable SFTS layer and replaceable PSH layer of MiS could be easily assembled or disassembled. The dual‐layered structure enables MiS a stable tactile sensing capability when the bottom PSH layer needs to be frequently replaced during wound management. Considering the rapidly increasing needs of wearable tactile sensors for paralyzed people, MiS demonstrates a possibility of wearing tactile sensors on the wounded skin for consistent tactile sensing and smart/expedited wound care. The future endeavor of connecting the tactile sensing capability of MiS to the peripheral neural network may also become possible.

## 3 Conclusions

In summary, MiS demonstrates a potential comprehensive solution to both structural and functional losses of skin and affiliated tissue due to severe wound, establishing a smart wound care strategy consisting of instant monitoring of wound conditions and responsive treatment, expedited healing performance, and multi‐tactile sensing reconstruction. Multiple functions of MiS were achieved by a novel versatile PSH film, acting as wound‐contacting surface and electrodes in SFTS sensor that possesses high‐loss factor viscoelasticity and self‐adaptive adhesiveness, high exudate absorbability, electrical conductivity, and broad‐spectrum antimicrobial activity. MiS revealed the ability to promote angiogenesis, epidermis formation, collagen deposition, and accelerate the wound healing process, as well as rapid, stable and multi‐modal sensation to the changes in wound conditions including pressure, temperature, exudate amount, joint motion or skin displacement. MiS therefore enables active diagnosis of wound conditions and instant therapeutic treatment. In addition, MiS was able to continuously detect the location, intensity, and trajectory of tactile signals, enabling a multi‐touch human‐machine interface controlling an external robotic arm.

We believe that MiS is the first reported ionic skin with the reparative capabilities of tactile sensing, complex wound monitoring and healing management. MiS demonstrated a great potential for skin regeneration and wound treatment at infected and chronic wound sites, particularly for patients with sensory disorders and/or dyskinesia either due to a preexisting condition or severe wound, by restoring haptic sense and operating capability. This potential opens a new avenue for future work in smart wound care solutions and related wound management materials or devices, including active and intelligent solutions to intervene inflammation and infection progress, management of extra‐large area wound bed, high‐quality regeneration of full‐thickness skin wound, and high‐fidelity tactile sensibility restoration.

## 4 Experimental Section

##### Preparation of MiS

Waxy starch (amylopectin>90%) was purchased from Qinhuangdao Lihua Starch Co., Ltd. (China). Ammonium persulfate, *N,N*′‐methylene bisacrylamide, and *N,N,N′,N′*‐tetramethylethylenediamine were purchased from Shanghai Aladdin Reagent Co., Ltd. (Shanghai, China). For preparation of the GPAH, a fixed water‐to‐starch mass ratio of 5 (for example, 1 g deionized water added with 0.2 g starch) was used, and the crosslinker Ca(NO_3_)_2_·4H_2_O was dissolved in the water at a mass concentration of 33.3%. The mixture was stirred at 60 °C to obtain a viscous GPAH. The PAM pre‐gel was prepared by adding 23 wt% acrylamide (Shanghai Macklin Biochemical Co., Ltd., China), 0.04 wt% of ammonium persulfate, 0.015 wt% of *N,N*′‐methylene bisacrylamide, and 0.062 vol% *N,N,N′,N*′‐tetramethylethylenediamine to deionized water under stirring at 37 °C for 3 min. The PAM/GPAH pre‐gel was mixed at mass ratio of 1:1 and stirred at 60 °C for 30 min in order to prepare PSH. To obtain PSH with specific geometries for various tests, the gel was cast into a container‐like petri dish to form a film of the desired thickness, and the gel film could be cut into the desired geometry and sizes. For example, samples with size of 14 × 14 × 2 mm was used for animal study. Disks (11 cm in diameter and 3 mm thick) were used for tactile sensing test.

Components A and B of Ecoflex 00–20 (American Smooth‐on Inc., USA) were mixed thoroughly (A:B = 1:1, v/v) for 3 min and poured into a mold with expandable polyethylene (as a separator between Ecoflex and PSH) at the bottom, followed by incubation for 5 h at 37 °C. The size of mold can be selected depending on specific applications. For example, tactile sensing test samples are 11 cm in diameter and 4 mm thick. Samples for joint motion monitoring are 48 × 8 × 3 mm large. After Ecoflex was cured, four strips of PSH electrode was adhered to its surface in square geometry (labeled as E1, E2, E3, and E4, Figure 1 to form a SFTS sensor.

At last, the SFTS was integrated with self‐adhesive PSH to form MiS. The bottom PSH base and top PSH electrodes could be wire‐connected with an oscilloscope or other signal acquisition device for detection of various signals.

##### Characterization of Physical and Chemical Properties of PSH

For the swelling test, PAM and PSH samples (14 mm in diameter, 2 mm thick), as well as sterile medical gauze (14 mm in diameter, 0.5 mm thick, AKX274, Tongda, China) and Tegaderm film (14 mm in diameter, 0.1 mm thick; Naxcare 3M, USA) were completely dried and immersed in deionized water at room temperature. At specified time intervals, the samples were retrieved and weighed after wipe of water from the sample surface. The water absorptivity of the materials was defined using the following Equation (1)

(1) $$\mathrm{Degree}\;\mathrm{of}\;\mathrm{swelling}\;\;\left(\%\right)=\frac{W_{i}-W_{0}}{W_{0}}\;\times 100\%$$

where *W*_i_ and *W*_0_ represent the weights of the swollen and dried sample, respectively.

The water vapor transmission rate (WVTR) of PAM and PSH was measured according to ASTM standard E96‐00, in comparison with gauze and Tegaderm film. After filling with 30 mL of water, the nozzle of a 50 mL centrifuge tube was sealed with PAM, PSH, gauze (1 mm thick) or Tegaderm film (0.1 mm thick), and the tube was placed in an incubator at 37 °C and 50% relative humidity for 1 day. The WVTR was defined by the following Equation (2)

(2) $$\mathrm{WVTR}\;\;\left(g\;\mathrm{per}\;\mathrm{day}\;\;m^{2}\right)=\frac{W_{0}-W_{i}}{t\times B}\;$$

where *t* is the test time, *B* is the nozzle area of the centrifuge tube, and *W*_0_ and *W*_i_ are the weights of the centrifuge tube containing water before and after water permeation in an incubator, respectively.

The tensile tests were performed on PAM and PSH samples (standard dumbbell shapes, 30 × 2 × 2 mm in length, width, and thickness) by a universal mechanical tester (HY‐0580, Shanghai Hengyi Co., Ltd., China) at a stretching speed of 100 mm min^−1^. Tensile strength, and elongation at break of samples were obtained. For the cyclic tensile tests, PAM or PSH samples were stretched to a strain of 100% and then unloaded back to 0. This cycle was repeated 200 times in order to determine tensile and recovery properties. Each mechanical test was repeated at least three times with distinct samples.

The rheological properties of the PAM and PSH samples (20 mm in diameter and 1‐ mm thick) were studied using a rheometer (AR2000, TA Instruments, USA). Frequency sweep tests were performed from 0.1 to 50 Hz at 37 °C and 1% strain. Three important parameters were determined: storage modulus (*G*′), loss modulus (*G*″), and loss factor (tan *δ* = *G*″/*G*′).

The adhesiveness of PAM and PSH to fresh porcine skin was determined using the previously described method^[^ ^27^ ^]^ based on ASTM standard C907‐17. Fresh porcine skin was flattened and fixed on both the upper and lower cross‐heads in a universal mechanical tester. The sample (14 mm in diameter and 1 mm thick) was placed over the porcine skin fixed on the lower cross‐head, and the upper cross‐head was lowered until the upper porcine skin fully contacted the sample to generate a small compression force of 1 N. When the force was relaxed to 0, the cross‐heads were separated at a speed of 60 mm min^−1^, and the stress‐displacement curve was recorded. Interfacial strength was defined as the maximum stress observed during the separation process. Interfacial toughness was defined as the amount of energy required to detach a unit area of hydrogel (1 mm thick) from the porcine skin and calculated by integrating the stress‐displacement curve. Three distinct samples were tested for all groups. The adhesiveness of swelled PAM and PSH to fresh porcine skin was also measured with the same experimental protocol.

##### Cytocompatibility Test

Since Ecoflex owns excellent cytocompatibility,^[^ ^86^ ^]^ the cytocompatibility of MiS was evaluated on PSH, which contacts with wound bed, by culturing NIH/3T3 fibroblasts and HUVEC in the hydrogel extracts according to ISO 10993–5. Cells were all purchased from the Stem Cell Bank of the Chinese Academy of Sciences. Hydrogel samples (14 mm in diameter and 2 mm thick) were sterilized by UV light for 48 h and immersed in Dulbecco's modified Eagle medium (DMEM) containing 10% v/v fetal bovine serum (FBS) and 1% v/v penicillin–streptomycin solution for 24 h at a ratio of 0.1 g mL^−1^ (additional supplementation of the medium was consumed by hydrogel swelling). Then the extracts were collected and filtered through a 0.22 µm membrane (Millex‐GP; Millipore, USA) to obtain the test medium before cell tests. Regular cell culture medium, consisting of DMEM with 10% v/v FBS and 1% v/v penicillin–streptomycin solution, was used for the control group. Cells were seeded in 96‐well plates at a density of 3500 cells/well. After 24 h, the regular medium was replaced with 100 µL of the hydrogel extracts, and after 1 and 3 days, cell viability was measured using a Cell Counting Kit‐8 (CCK‐8, Dojindo Molecular Technologies, Inc., Japan). Optical density (OD) values were measured at 450 nm using a microplate reader (PowerWave X, BioTek Instruments, USA). Each sample was tested in triplicate, with relative growth rate calculated according to the following formula (Equation (3))

(3) $$\mathrm{Relative}\;\mathrm{growth}\;\mathrm{rate}\;\;\left(\%\right)=\frac{OD_{i}}{OD_{0}}\;\times 100\%$$

where OD_i_ is the OD value on days 1 and 3 for the extract group or day 3 for the control group, and OD_0_ is the OD value on day 1 for the control group.

For imaging assay, NIH/3T3 fibroblasts or HUVEC were seeded in 24‐well plates at a density of 10 000 cells/well, and following adherence to the plate for 24 h, the cell culture medium was replaced with hydrogel extracts, and the cells were cultured for 1 and 3 days. For the control group, regular cell culture medium was used. The cells were then stained using a LIVE/DEAD viability kit (Thermo Fisher Scientific, USA) and imaged by fluorescence microscopy (NR3831001630, Carl Zeiss, Germany). Three replicates were tested for all groups.

##### Hemolysis Test

The hemolytic potential of MiS was evaluated by measuring the hemolysis rate of PSH according to ASTM standard F‐756‐08, and compared with PAM. PSH or PAM (14 mm in diameter and 2‐mm thick) were immersed in saline at 0.1 g mL^−1^ for 24 h at 37 °C (additional saline supplementation was consumed by hydrogel swelling). Only saline were used in the negative control group and distilled water in the positive control group. Anticoagulated rabbit heart blood was diluted with saline. The hydrogel extracts (4 mL) were added to a centrifuge tube and placed in a water bath for 30 min at 37 °C, after which 80 µL of diluted rabbit blood was added to each tube, gently mixed, and incubated in a water bath for 1 h at 37 °C. The tubes were then centrifuged at 3500 rpm for 15 min, and the OD of the supernatant was measured at 545 nm using a microplate reader (PowerWave X, BioTek Instruments, USA). Three replicates were tested for all groups. Hemolysis rate was calculated according to the following formula (Equation (4))

(4) $$\mathrm{Hemolysis}\;\mathrm{rate}\;\;\left(\%\right)=\frac{OD_{i}-OD_{\mathrm{nc}}}{OD_{\mathrm{pc}}-OD_{\mathrm{nc}}}\;\times 100\%$$

where OD_i_ is the OD value for the extract group, OD_nc_ is the OD value for the negative control, and OD_pc_ is the OD value for the positive control.

##### Antimicrobial Test

The antimicrobial activities of the PSH in MiS were evaluated against three bacteria (*Staphylococcus aureus*, *Escherichia coli*, and *Pseudomonas aeruginosa*) and one fungus (*Candida albicans*) using the direct contact method,^[^ ^37,893938^ ^]^ and were compared to PAM. PSH, PAM, or sterile medical gauze samples were tiled in a 24‐well plate to a thickness of 2 mm, with the empty well as a blank control. The number of bacteria or fungus initially seeded was determined as respective controls, with 100 µL of bacterial or fungal suspension at 1 × 10^6^ CFU mL^−1^ added to each well and cultured for 4 h under a humidified atmosphere of 5% CO_2_ at 37 °C. After prescribed time periods, the bacterial or fungal suspension and materials were transferred to a centrifuge tube, the 24‐well plate was washed three times with saline, and the wash was similarly transferred to a centrifuge tube. The bacterial or fungal solution and material were sonicated for 15 min, followed by serial dilution and transfer of 100 µL of diluted bacterial or fungal solution to an agar plate for culture for 24 h. Colonies were recorded and photographed at the end of the culture, with each sample tested in triplicate. Antimicrobial activity was calculated according to the following formula (Equation (5))

(5) $$\begin{array}{ccc} & & \mathrm{Antimicrobial}\;\mathrm{rate}\;\left(\%\right)=\\ & & \;\frac{\mathrm{cell}\;\mathrm{count}\;\mathrm{of}\;\mathrm{the}\;\mathrm{control}-\mathrm{cell}\;\mathrm{count}\;\mathrm{on}\;\mathrm{hydrogels}}{\mathrm{cell}\;\mathrm{count}\;\mathrm{of}\;\mathrm{the}\;\mathrm{control}}\;\times 100\% \end{array}$$

For morphological observation, microbes were seeded on the surface of the material and cultured for 4 h. After that, the microbes were fixed with 4% paraformaldehyde, and were dehydrated in gradients with alcohol solutions. The samples are then placed in a CO_2_ critical point freeze dryer (CPD030C, LEICA, Germany) for drying, and were sputter‐coated for 60 s using an ion‐sputter coating instrument (SC7620, Quorum, England) for morphological observation by scanning electron microscope (SEM, Quanta 250, FEI, USA).

##### Animal Study

All animal procedures were performed according to the protocol approved by the Ethics Committee of Soochow University (Approval No. ECSU‐2019000160). Institute of Cancer Research mice (male, ≈30 g) were obtained from the Laboratory Animal Center of Soochow University. The full thickness‐skin defects were treated with MiS, with commercial sterile wound dressing (SWD, HN‐001, Hainuo, China) and PAM as controls (*n* = 8/group). PAM was used because it is an important component in the MiS, and has also been used as wound dressing.^[^ ^87^ ^]^ All surgical procedures were performed under aseptic conditions. Mice were anesthetized by intraperitoneal injection of ketamine hydrochloride/xylazine (10 µL g^−1^ body weight). A round full‐thickness skin defect of 10 mm in diameter was made on the back of the mouse and rinsed with saline, after which SWD, PAM, or MiS was placed on the wound and fixed with medical tape at the rim of devices. Dressings were changed daily, and the wound area was measured on days 0, 5, 10, and 15. Wound contraction was determined using the following formula (Equation (6))

(6) $$\mathrm{Wound}\;\mathrm{contraction}\;\;\left(\%\right)=\frac{A_{0}-A_{i}}{A_{0}}\;\times 100\%$$

where *A*_0_ represents the wound area on day 0, and *A*_i_ is the wound area on days 5, 10, or 15.

To evaluate skin regeneration in that area, the wound area tissue from mice collected on day 15 (*n* = 5/group) was fixed with 10% formaldehyde solution for 12 h, embedded in paraffin, and cross‐sectioned to 6 µm thick slices. Hematoxylin & Eosin (H&E), Masson, and CD31‐specific immunohistochemical staining were performed on the skin sections, and the stained sections were observed by microscopy.

##### Strain, Temperature, and Exudate‐Sensing Tests

The responses of MiS (50 × 10 mm) to strain, temperature, and exudate stimuli were determined according to changes in current through the samples, which was measured using a two‐electrode method. A platinum‐wire electrode was connected to the both ends of bottom PSH (2 mm in thickness) of MiS, and constant voltage of 3 V was applied to the MiS through a constant voltage power supply (IT6833, ITech, China). A multimeter (17B, Fluke, Everett, WA, USA) was used to monitor current through MiS. The required tensile strain was applied to the MiS through a universal mechanical tester. Meawhile, the MiS was attached to the back of the human hand to monitor metacarpophalangeal joint movements. The human pilot study involved one healthy volunteer. The study was approved by the Ethics Committee of Soochow University (Approval No. SUDA20200510A01). The participant agreed and signed to the consent form to allow the experiment procedure. The temperature and exudation sensing capabilities of MiS were measured using an ex vivo model of porcine full‐thickness skin defect (20 × 20 mm). For the temperature sensing test, the skin around wound was heated and the change in resistance of MiS with temperature was recorded within 32–42 °C. For exudation sensing measurement, gauze fully absorbed with saline was inserted in the wound at 35 °C or 42 °C, and was connected with a saline reservoir for supplementing saline (with temperature identical to wound) in the gauze to simulate the continuous exudation at the wound site. After contact with the wound with exudation, the change in resistance of the MiS was monitored.

The responsiveness of MiS was calculated and expressed by the △*R*/*R*_0_ ratio, where *R*_0_ and △*R* are original resistance and the change in resistance when the stimuli (temperature, strain, exudation, etc.) were applied, respectively.

##### In Vitro Drug Release Test

To test the pH‐responsive drug release property of MiS, bovine serum albumin (BSA, Sigma‐Aldrich, USA) was used as a model drug and added to PSH (14 mm in diameter and 2‐mm thick) at a mass fraction of 1% during preparation. The BSA‐loaded PSH samples were placed into 5 mL of PBS at various pH values (pH = 4, 5, 6, 7, 8, 9, and 10) at 37 °C. At predetermined time intervals, 5 mL of immersion solution was transferred, and 5 mL of fresh PBS at a different pH was added. The quantity of BSA in the immersion solution was determined using the BCA protein assay kit (P0012S, Beyotime, China). The absorbance was measured at 562 nm in a microplate reader (PowerWave X, BioTek Instruments, USA).

##### Tactile Sensing and Robotic Control Tests

To demonstrate the tactile sensing capability of the MiS, 4 × 4 test lattices (20 × 20 mm each lattice) were labeled on the top surface of MiS (110 mm in diameter and 4‐mm thick). The flexible PSH electrodes (E1, E2, E3, and E4) were connected to an oscilloscope (DSOX3032T, Keysight, USA) with wires. Upon finger contact with the test lattices, voltages V (E1), V (E2), V (E3), and V (E4) for E1, E2, E3, and E4 were recorded through the oscilloscope, and two voltage ratios [V (E3) / V (E1) and V (E4) / V (E2)] were used to represent the position of the contact point of the finger with each test lattice. Measurements were repeated 15 times for each test lattice, and the average value for the standard contact point in the square area was calculated. The test lattices were randomly touched with fingers to verify error ranges for the standard contact points. The effect of force on the tactile performance of the MiS was assessed by contacting and pressing the lattice No. 11 with different force (0.5, 1, or 1.5 N) and recording voltage and voltage ratios for E1, E2, E3, and E4 under different force values.

To demonstrate how MiS works as a human‐machine interface to use the tactile signals for robotic control, a system that included the MiS, a signal‐acquisition system (NI USB‐6009), a computer, a drive system (UR5), and a robotic arm (Universal robot) was built according to the previous work.^[^ ^20^ ^]^ The capability of MiS, as a flexible tactile‐controlled human‐machine interface, to control robotic arm was evaluated by touching lattices No. 4, 7, 10, 13 in sequence. The tactile signals were transferred into voltage signals by MiS and collected by the signal‐acquisition system, which were used to control the robotic arm by the drive system.

##### Statistical Analysis

All the results were reported as a mean with standard deviation. Sample size (*n*) of independent repeated experiments for each statistical analysis was given in the figure legends. Statistical differences between two groups were determined by two‐tailed unpaired Student's *t*‐test using GraphPad Prism 6 (GraphPad Software, USA). Differences were considered significant at *p* < 0.05.

## Conflict of Interest

The authors declare no conflict of interest.

## Supporting information

Supporting Information

Supplemental Movie 1

## Data Availability

The data that support the findings of this study are available from the corresponding author upon reasonable request.
