# Supplementary material for: Ultra‐Conformable Ionic Skin with Multi‐Modal Sensing, Broad‐Spectrum Antimicrobial and Regenerative Capabilities for Smart and Expedited Wound Care
Source: Adv Sci (Weinh). 2021 Feb 15;8(9):2004627. doi: 10.1002/advs.202004627 (PMC8097371; doi:10.1002/advs.202004627)
Supplement: Supplementary file 1 — Supporting Information [file ADVS-8-2004627-s002.pdf]

© 2021 Wiley-VCH GmbH

ADVANCED SCIENCE
Open Access

# Supporting Information

for *Adv. Sci.,* DOI: 10.1002/advs.202004627

**Ultra-Conformable ionic skin with multi-modal sensing, broad-spectrum antimicrobial and regenerative capabilities for smart and expedited wound care**

Xiao Lin, Yuxuan Mao, Peng Li, Yanjie Bai, Tao Chen, Kang Wu, Dandan Chen,
Huilin Yang, Lei Yang*

**Supplementary methods**

*Fourier Transform Infrared (FTIR) and scanning electron microscopy (SEM) analyses.* FTIR spectra of the PAM, GPAH, and PSH were recorded in a range of 500 $cm^{-1}$ to 3800 $cm^{-1}$ using FTIR spectroscopy (Nicolet 6700, Thermo Fisher Scientific, USA). The microstructure of the surface of freeze-dried PAM and PSH was observed by SEM (JSM-7100F, JEOL, Japan). The SEM samples were sputter-coated for 60 s using an ion-sputter coating instrument (SC7620, Quorum, England).

*Measurements of the adhesiveness of PSH partially swelled with saline.* In order to evaluate the effect of normal perspiration on the adhesiveness of PSH to skin, partially swelled PSH was prepared by absorbing 50 wt % of saline. The interfacial strength and interfacial toughness of partially swelled PSH to fresh porcine skin was measured with the same experimental protocol of test on as-prepared PSH.

*Measurements of the water absorption of PSH at different pH value.* PSH samples were immersed in PBS at various pH values (pH = 4, 7, and 10) at 37 °C. At 12 h, the samples were retrieved and weighed after wipe of water from the sample surface. The amount of water absorbed in the materials was defined using the following equation:

$$\text{Water absorption ratio (\%)} = \frac{W_i - W_0}{W_0} \times 100\%$$

where $W_i$ and $W_0$ represent the sample weight after immersed in PBS for 12 h and the sample theoretical dry weight, respectively.

*Measurements of electrical conductivity properties of PSH.* The PSH was cut into strips (30 × 8 × 3 mm in length, width, and thickness) and connected with two platinum wires to form a circuit. A constant voltage of 3 V was applied to the circuit through a constant voltage power supply (IT6833, ITech, China). The current in the circuit was measured by a multimeter (17B, Fluke, USA) to calculate the resistance of PSH.

To evaluated the self-healing capability of conductivity, the PSH conductor was cut in half and re-connected. The variation in the current before cutting and after re-connecting were measured by a multimeter and also demonstrated by the brightness of a LED light that was connected in series with PSH. The change in the current was expressed by the $I/I_0$ ratio, where I and $I_0$ are the current through damaged (either separated or re-connected) and intact hydrogel samples, respectively.

In order to evaluated the effect of perspiration-caused NaCl retention on the electrical property of PSH, the conductivity of PSH containing 0.9 wt % of NaCl was also measured.

*Measurement of gauge factor of MiS in response to tensile strain.* A platinum-wire electrode was connected to the PSH base (35 × 8 × 3 mm in length, width, and thickness) of MiS, and a constant voltage of 3 V was applied by a constant voltage power supply (IT6833, ITech, China). MiS were stretched to 100, 200, 300 and 400% strains and the relative resistance changes were measured by a multimeter (17B, Fluke, USA). The gauge factors are calculated based on the formula (7):

$$\text{Gauge factor} = \frac{\Delta R}{R_0 \times \varepsilon} \quad (7)$$

where $R_0$ is the resistance when the strain is 0%; $\Delta R$ is the changes in the resistance when the strain of MiS is 100, 200, 300 and 400%, respectively; $\varepsilon$ is the strain of MiS.

*Measurement of strain sensing capability of MiS in a rat wound model.* In order to evaluate the strain sensing on a wound *in vivo*, MiS (30 × 10 × 3 mm) was attached on the rat knee joint with and without a full-thickness wound to monitor the cyclic bending of knee joint. The study was approved by the Ethics Committee of Soochow University (Approval No. SUDA20201111A01). The test was carried out under anesthesia in rats.

*Measurement of response time to temperature change.* Two same *ex vivo* porcine skin wound models were used for the test. The two wound models were incubated at 32°C and 42°C, respectively. After stabilized on the wound model incubated at 32°C, the MiS was rapidly moved onto the wound model incubated at 42°C and the change of $\Delta R/R0$ value with time was

monitored. The change of △R/R0 value was also monitored after moving MiS from the wound model incubated at 42℃ onto the wound model incubated at 32℃. The PSH base of MiS is 48 × 19 × 1.5 mm in length, width, and thickness.

*Tactile sensing test in a rat wound model*: To demonstrate the tactile sensing capability of the MiS on a wound *in vivo*, a MiS with 2 × 2 test lattices (20 × 20 mm each lattice) was appllied on a full-thickness skin defect with a diameter of 30 mm on the rat back. Voltages V (E1), V (E2), V (E3), and V (E4) for E1, E2, E3, and E4 were recorded using the oscilloscope, and two voltage ratios [V (E3) / V (E1) and V (E4) / V (E2)] were used to represent the position of the contact point of the finger with each test lattice. The study was approved by the Ethics Committee of Soochow University (Approval No. SUDA20201111A01). The test was carried out under anesthesia in rats.

*Statistical analysis*: All the results were reported as a mean with standard deviation. Sample size (n) of independent repeated experiments for each statistical analysis was given in the figure legends. Statistical differences between two groups were determined by two-tailed unpaired Student's t-test using GraphPad Prism 6 (GraphPad Software, USA). Differences were considered significant at $p < 0.05$.

**Supplementary text**

**Cross-linking mechanism of PSH**

The formation of covalent bonds between acrylamide monomers is the result of free radical polymerization.[88, 89] The physical cross-linking of starch molecules in GPAH is likely mediated through ligand binding between amylopectin hydroxyl groups and positively charged $Ca^{2+}$.[90-92] The FTIR of PSH (Figure S1) revealed a peak at 3220 $cm^{-1}$ corresponding to an -$NH_2$ group and another one at 1616 $cm^{-1}$ corresponding to the bending vibration of N-H in the PAM. Additionally, the characteristic peak at 3440 $cm^{-1}$ represented the absorption peak of -OH in GPAH. These characteristic peaks of PAM and GPAH were observed within the PSH infrared spectrum but no new characteristic peaks were generated. This result indicates that GPAH and PAM formed an interpenetrating network but no additional chemical reaction between molecules occurred. Moreover, SEM images (Figure S2) of the freeze-dried hydrogel samples show that the average pore size of the PSH was greater than that of PAM, indicating that the addition of GPAH to PAM decreased the cross-linking density of PAM.

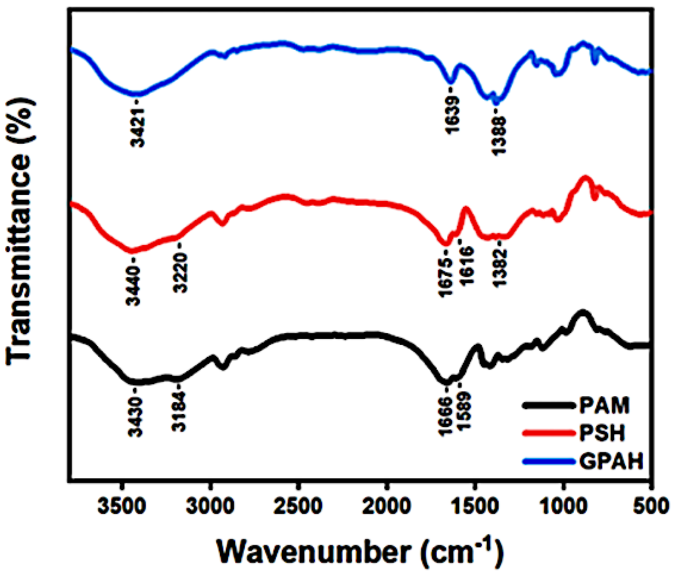


**Figure S1.** FTIR spectra of PAM, PSH and GPAH.

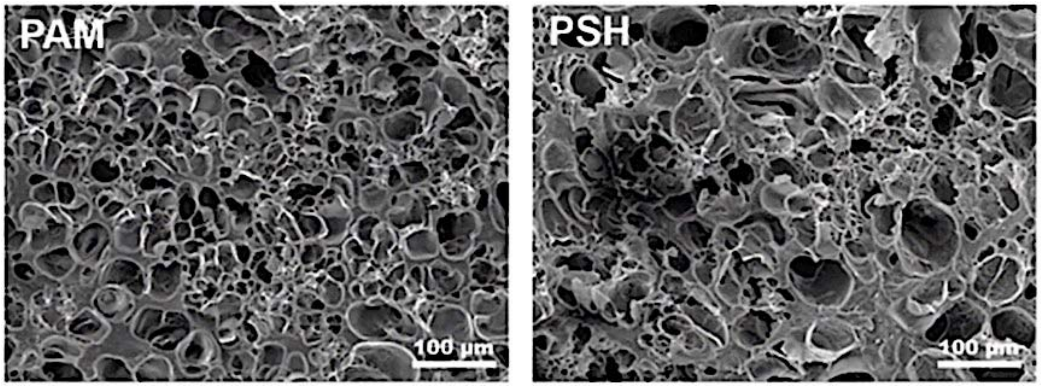


**Figure S2.** SEM images of freeze-dried PAM and PSH. Both PAM and PSH demonstrated homogeneous porous structures, but the average pore size of PSH is greater than that of PAM.

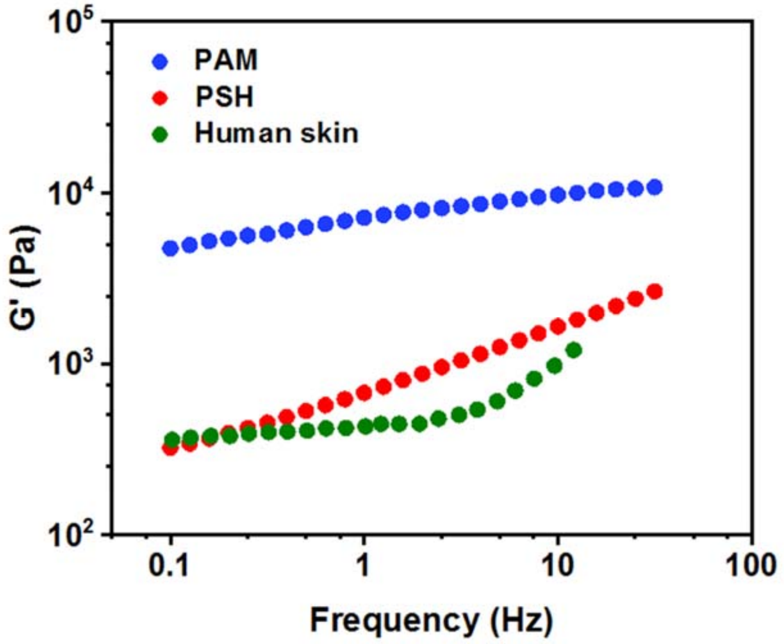


**Figure S3.** Dependence of storage modulus of hydrogels and human skin [32] on the frequency of oscillation.

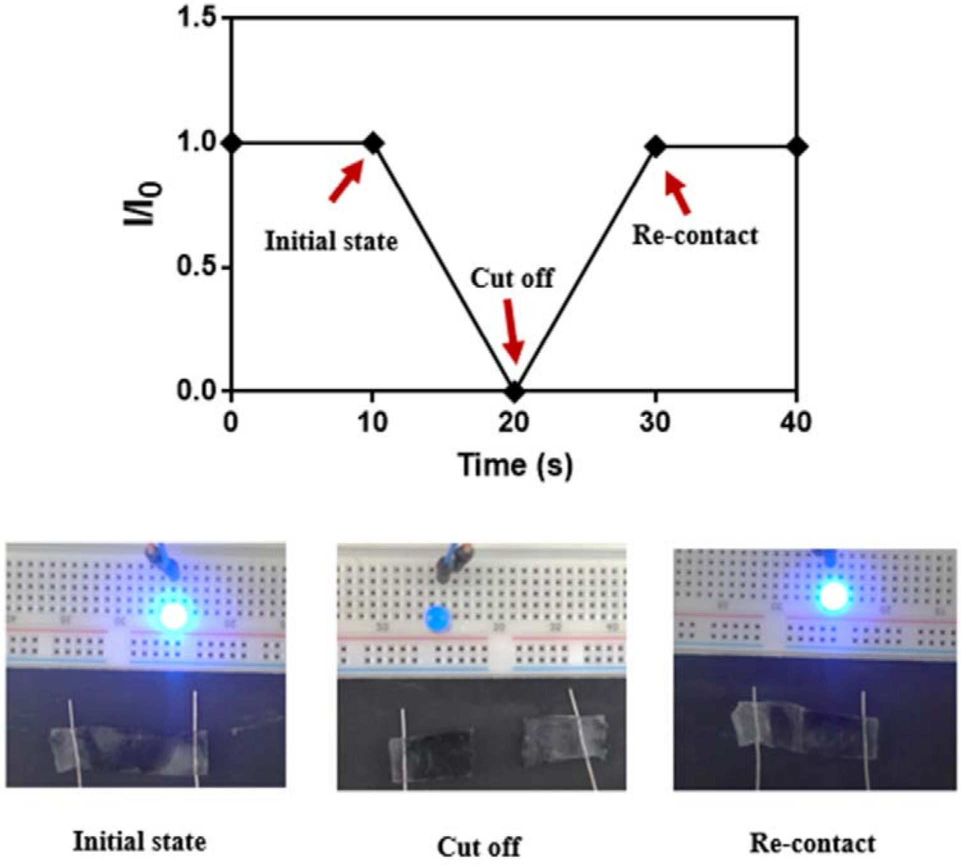


**Figure S4.** Changes of $I/I_0$ values when PSH was cut in half and re-connected at the damaged edges. The variation in current was also demonstrated by the brightness of a LED light that was connected in series with PSH.

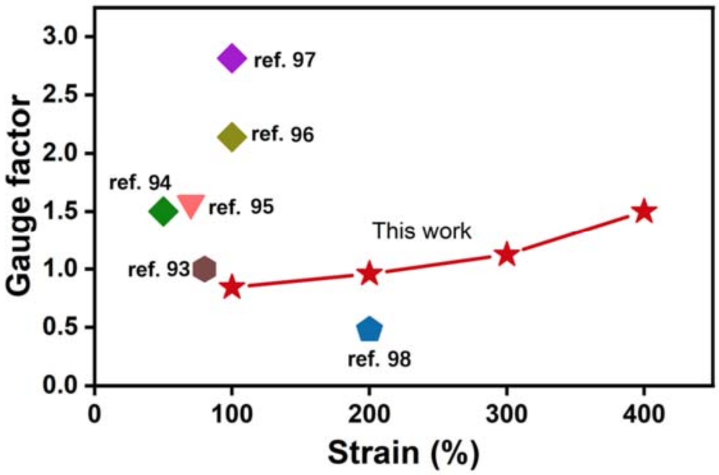


**Figure S5.** Comparison of gauge factor of PSH with those of previously reported hydrogel strain sensors. The gauge factors of PSH at various applied strains were demonstrated. The gauge factors from references are the highest values of what reported in the studies.[93-98]

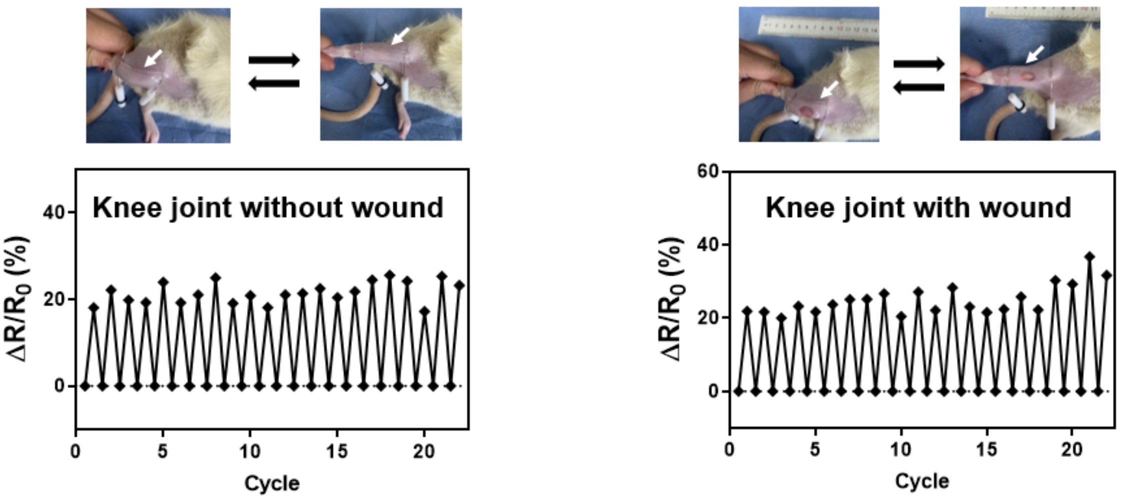


**Figure S6.** Performance of the PSH layer of MiS adhered on a rat joint (with or without wound) as a strain sensor for instantly detecting knee joint movements. White arrows indicate the PSH layers.

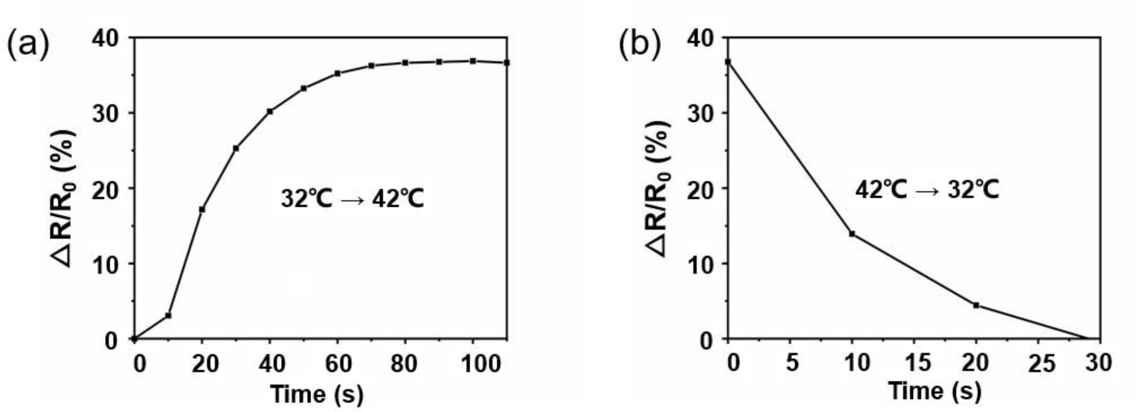


**Figure S7. Response time of MiS to temperature change in an *ex vivo* porcine skin wound model.** (a) Variation in △R/R0 with time after moved a MiS from the wound model incubated at 32℃ onto the wound model incubated at 42℃. (b) Variation in △R/R0 with time after moved a MiS from the wound model incubated at 42℃ onto the wound model incubated at 32℃. The PSH base of MiS is 48 × 19 × 1.5 mm in length, width, and thickness.

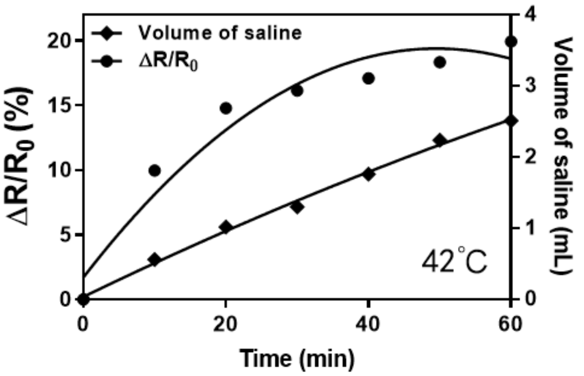


**Figure S8.** Variation in △R/R0 and saline volume absorbed into a MiS sample with time during contacting with an *ex vivo* wound for 60 min at 42°C. The volume of PSH base of MiS is 1 $cm^3$, and the contact area is 5 $cm^2$.

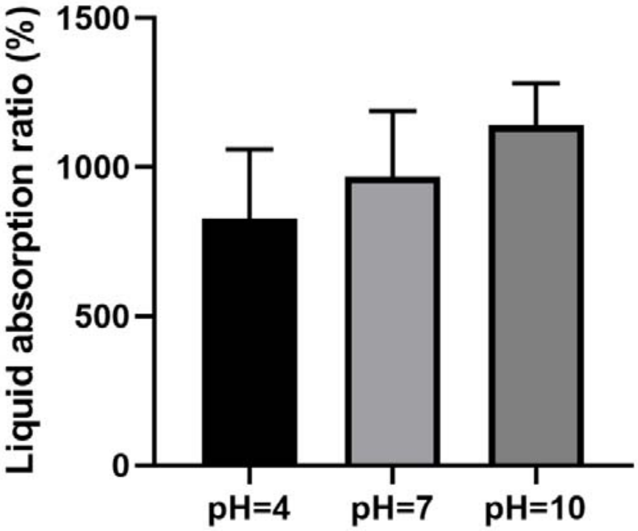


**Figure S9.** The PBS absorption ratio of PSH after immersed in PBS at various pH values (pH = 4, 7, and 10) at 37 °C for 12 h. Data=mean ± standard deviation (n=3).

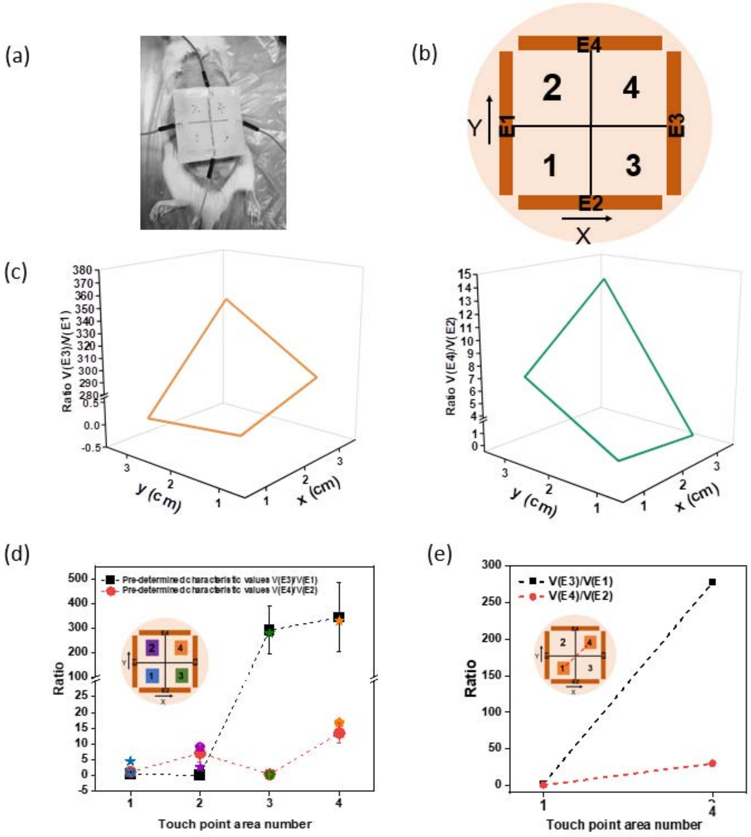


**Figure S10. Multi-tactile sensing capability of MiS evaluated by a rat wound model.** (a) Photograph of a MiS covered the skin wound on the back of a rat for testing the sensing of complex tactile signals. (b) Schematic illustration of the configuration of the top surface of MiS. The area within the four electrodes was divided into 4 lattices for location differentiation. (c) Voltage ratios of E3/E1and E4/E2 (averaged from 15 independent tests) with 4 testing points. (d) All pre-determined characteristic V(E3)/V(E1) and V(E4)/V(E2) values (means and standard deviations of 15 independent tests) of all 4 lattices, as well as the values in the reliability test. Inset: Schematic illustration of four points selected for reliability test. (e) Variation in V(E3)/V(E1) and V(E4)/V(E2) values when a finger touched lattices No. 1 and 4 in sequence.

**Table S1.** The water vapor transmission rate (WVTR) of PAM, PSH and several commercial wound dressings.

| Sample | WVTR (g/m$^2$.day) |
|---|---|
| Honey Pads* | 700 |
| Tegaderm$^{TM}$ Film** | 495 |
| Silicone Scar Sheet *** | 255 |
| Gauze | 3548 |
| PAM | 740 |
| PSH | 1442 |

*Honey Pads: Sterile Manuka Honey Adhesive Pads, CVS Health, USA

** Tegaderm$^{TM}$ Film: Nexcare Tegaderm waterproof transparent dressing, 3M, USA

*** Silicone Scar Sheet: Silicone Scar Sheet, Scar Away, USA

**Movie S1 (separate file)**

Demonstration of the MiS, as a flexible human-machine interface, accurately controlling a robotic arm by sensing tactile signals on its top surface. The robotic system includes the MiS, a signal-acquisition system, a computer, a drive system, and the associated robotic arm. Tactile signals were sensed by the top surface of MiS when a finger touch the lattices No. 4, 7, 10 and 13 in consecutive sequence. MiS generated characteristic electrical signals of V(E3)/V(E1) and V(E4)/V(E2) values. These signals were input to the computer for accurately controlling the movement and positions of the robotic arm.

**SI References**


[88] H. M. El-Din, S. G. Alla, A. W. El-Naggar, *J. Macromol. Sci. A* **2007**, *44*, 47.

[89] K. Ou, X. Dong, C. Qin, X. Ji, J. He, *Mater. Sci. and Eng. C* **2017**, *77*, 1017.

[90] W. Ciesielski, M. Krystyjan, *e-Polymers* **2009**, *9*, 1628.

[91] W. Ciesielski, P. Tomasik, *J. Inorg. Biochem.* **2004**, *98*, 2039.

[92] Y. Chen, C. Wang, T. Chang, L. Shi, H. Yang, M. Cui, *Starch-Stärke* **2014**, *66*, 149.

[93] C. Shao, M. Wang, L. Meng, H. Chang, B. Wang, F. Xu, J. Yang, P. Wan, *Chem. Mater.* **2018**, *30*, 3110.

[94] Z. Wang, J. Chen, L. Wang, G. Gao, Y. Zhou, R. Wang, T. Xu, J. Yin, J. Fu, *J. Mater. Chem. B* **2019**, *7*, 24.

[95] J. Huang, D. Li, M. Zhao, P. Lv, Q. Wei, *Cellulose* **2019**, *26*, 3401.

[96] C. Hu, Y. Zhang, X. Wang, L. Xing, L. Shi, R. Ran, *ACS Appl. Mater. & Inter.* **2018**, *10*, 44000.

[97] Z. Gao, Y. Li, X. Shang, W. Hu, G. Gao, L. Duan, *Mater. Sci. and Eng. C* **2020**, *106*, 110168.

[98] Y.-J. Liu, W.-T. Cao, M.-G. Ma, P. Wan, *ACS Appl. Mater. & Inter.* **2017**, *9*, 25559.
